# Continuous Hypoxic Culturing of Human Embryonic Stem Cells Enhances SSEA-3 and MYC Levels

**DOI:** 10.1371/journal.pone.0078847

**Published:** 2013-11-13

**Authors:** Elisa Närvä, Juha-Pekka Pursiheimo, Asta Laiho, Nelly Rahkonen, Maheswara Reddy Emani, Miro Viitala, Kirsti Laurila, Roosa Sahla, Riikka Lund, Harri Lähdesmäki, Panu Jaakkola, Riitta Lahesmaa

**Affiliations:** 1 Turku Centre for Biotechnology, Turku University and Åbo Akademi University, Turku, Finland; 2 Department of Oncology and Radiotherapy, Turku University Hospital, Turku, Finland; 3 Department of Information and Computer Science at Aalto University School of Science, Espoo, Finland; Kanazawa University, Japan

## Abstract

Low oxygen tension (hypoxia) contributes critically to pluripotency of human embryonic stem cells (hESCs) by preventing spontaneous differentiation and supporting self-renewal. However, it is not well understood how hESCs respond to reduced oxygen availability and what are the molecular mechanisms maintaining pluripotency in these conditions. In this study we characterized the transcriptional and molecular responses of three hESC lines (H9, HS401 and HS360) on short (2 hours), intermediate (24 hours) and prolonged (7 days) exposure to low oxygen conditions (4% O_2_). In response to prolonged hypoxia the expression of pluripotency surface marker SSEA-3 was increased. Furthermore, the genome wide gene-expression analysis revealed that a substantial proportion (12%) of all hypoxia-regulated genes in hESCs, were directly linked to the mechanisms controlling pluripotency or differentiation. Moreover, transcription of *MYC* oncogene was induced in response to continuous hypoxia. At the protein level MYC was stabilized through phosphorylation already in response to a short hypoxic exposure. Total MYC protein levels remained elevated throughout all the time points studied. Further, MYC protein expression in hypoxia was affected by silencing HIF2α, but not HIF1α. Since MYC has a crucial role in regulating pluripotency we propose that induction of sustained MYC expression in hypoxia contributes to activation of transcriptional programs critical for hESC self-renewal and maintenance of enhanced pluripotent state.

## Introduction

Pluripotent human embryonic stem cells (hESCs) are derived from pre-implantation blastocysts. As early embryonic development takes place in low oxygen environment [1], [2], continuous hypoxic culturing of hESCs more closely resembles their physiologic environment than culturing them in normoxia. This is supported by results showing that hypoxic growth conditions (less than 5% O_2_) prevent spontaneous differentiation, reduce chromosomal aberrations, maintain active X-chromosome state and enhance hESCs self-renewal [3]–[7]. Furthermore, many recent studies have shown that low oxygen facilitates directional differentiation and helps to maintain the multipotency of stem and progenitor cells [8]–[11]. Subsequently, the reduced oxygen levels significantly increase production of desired cell populations from induced pluripotent stem cells (iPSCs) [12]–[15]. Moreover, low oxygen also potentiates generation of iPSCs from mouse and human fibroblasts [16]. Collectively, all available data suggest that hypoxia is important for the maintenance of undifferentiated cell state as well as unlocking the directional differentiation potential of stem cells. However, very little is known about the molecular mechanisms underpinning these processes.

In all cell types much of the hypoxic responses are mediated by hypoxia-inducible transcription factors (HIFs). HIFs are heterodimeric molecules consisting of HIFα and HIFβ subunits. The β-subunit is constitutively expressed whilst α-subunit is constitutively degraded in normally oxygenated cells. The Von Hippel-Lindau (pVHL) protein is an E3 ubiquitin ligase required for ubiquitination of HIFα once these proteins have physically interacted. Ubiquitination targets HIF-α to proteasomes and subsequent degradation [17]. The mechanism of cellular oxygen sensing is bound to this interaction, since the recognition and binding of pVHL to HIFα requires oxygen-dependent hydroxylation of conserved proline residues on HIFα by HIF prolyl hydroxylases (PHD1–3) [18]–[20]. In hypoxic conditions hydroxylation is inhibited leading to stabilization of HIFα and activation of oxygen-sensitive responsive transcriptional programs.

MYC is a pleiotropic transcription factor having thousands of binding sites throughout the genome regulating various cellular processes such as proliferation, growth, ontogenesis, transformation and differentiation. More importantly, MYC is essential for driving self-renewal and maintaining the pluripotent state of mouse embryonic stem cells (mESCs) as well as hESCs [21]–[23]. Further, overexpression of MYC effectively enhances reprogramming of differentiated somatic cells to iPSCs [24]. In addition, MYC is the key connecting point in protein interaction networks enriched in hESCs [25], [26].

Here we report detailed analysis of molecular and transcriptional responses of three hESC lines (H9, HS401 and HS360) to short (2h), intermediate (24 h) and prolonged (7d) continuous hypoxia (4% O_2_). Our data shows that culturing of hESCs in low oxygen activates transcriptional processes that support pluripotency and prevent differentiation. Furthermore, the results suggest that elevated MYC level is responsible for hypoxia-mediated maintenance of dedifferentiating regulatory programs in hESCs.

## Materials and Methods

### Ethics Statement

Ethics Committee of South-West Finland Hospital District has given us the permission to grow hESC lines. Research was carried out following the good scientific practice and guidelines of the National Advisory Board on Research Ethics.

### Cell Culture

Human ESC lines H9 and H7 [27] were obtained from WiCell, HS360 and HS401 [28] were obtained from Outi Hovatta (Karolinska Institute, Sweden). Stock cultures of hESCs were cultured on 0.1% gelatin (Sigma) coated plates on human foreskin fibroblasts (ATCC CRL-2429) having media composition; DMEM F12 (Stem Cell Technologies), 20% Serum Replacement, Knockout SR (Gibco), 2 mM Glutamax (Gibco), 1% Non-essential Aminoacids (Gibco), 48,5 U/ml Penicillin-Streptomycin (Gibco), 0.1 mM 2-mercaptoethanol (Gibco), 4 ng/ml rhFGF (R&D Systems). Media was changed daily. Cultures were divided by enzymatic passaging with Collagenase IV (Gibco 17104-019).

Embryonal carcinoma cell line 2102Ep [29] was obtained from Peter Andrews (University of Sheffield, UK). The cells were maintained in DMEM (Sigma) supplemented with 10% fetal calf serum (FCS) (PromoCell) and 2 mM l-glutamine (Sigma) and passaged using 0.05% trypsin-EDTA.

### Experimental Setup

Cells passaged with Collagenase IV were cultured on Matrigel (BD Biosciences) coated plates having mTeSR1 (StemCell Technologies) media. One day after seeding, 7 day time point plates were transferred to hypoxia incubator (Invivo2 400, Ruskinn technologies, UK), which allows media change and handling of cells to be performed under strictly controlled atmospheric conditions. In order to have sufficient amount of cells before collection, the 24 hour time point plates were cultured for 2 days and 2 h time point plates for 3 days in normoxia (21% O_2_ and 5% CO_2_) prior transfer to hypoxic culture conditions (4% O_2_ and 5% CO_2_). The low oxygen balanced media was changed to the plates immediately when cells were transferred into hypoxia incubator. The cells were fed daily with growth media balanced overnight in correct CO_2_ and O_2_ conditions for both hypoxia and normoxia cultured cells. Hypoxia treated cells were harvested in hypoxia incubator. After harvesting, the samples were frozen immediately with dry ice and stored at –80°C.

### Detection of Proteins

For protein analysis cells were harvested in SDS-Triton lysis buffer (50 mM Tris-HCl pH 7.5, 150 mM NaCl, 10 mM NaF, 0.5% Triton X-100, 1% SDS, 1 mM Na_3_VO_4_, 1 mM phenylmethylsulfonyl fluoride and Complete protease inhibitors) followed by addition of 6 x SDS buffer, sonication and boiling prior to loading. Protein concentrations were measured with Bio-Rad DC-protein assay prior to addition of 6 X SDS buffer and equal amounts of protein was loaded and run on SDS-PAGE in a mini-gel chamber (Bio-Rad) and transferred on Nitrocellulose-membrane (Bio-Rad). Western blot analyses with the specific antibodies were performed. Information on antibodies and dilutions used is provided in File S1. All primary antibody incubations were carried out overnight at +4°C followed by Horseradish peroxidase-conjugated secondary antibody treatment for one hour at room temperature. Proteins were detected with enhanced chemiluminescence reagent (Pierce).

### Exon Arrays

To study transcriptional changes, RNA was isolated from three different timepoints 2 h, 24 h and 7 days and from both O_2_ conditions. Experiment was repeated with three different hESC lines resulting 18 samples with three biological replicates for each timepoint and condition.

RNA was isolated from cell pellets with RNeasy Kit (Qiagen). To eliminate genomic DNA from RNA samples DNase I (Qiagen) digestion in the column was included. RNA concentration in the samples was measured with Nanodrop (Thermo Scientific). Samples were hybridized on GeneChip Human Exon 1.0 ST Arrays (Affymetrix) according to manufacturer’s protocol in the Finnish Microarray and Sequencing Centre, at Turku Centre for Biotechnology, Finland.

### Exon Array Data Analysis

The data (available at database GEO: GSE35819) was pre-processed with Chipster open source analysis platform (http://chipster.csc.fi/) using RMA method. The probes were re-annotated using the custom CDF package provided by Brainarray [30] and the expression signals were summarized at gene level based on Entrez gene annotations. The downstream data analysis was carried out with R/Bioconductor software [31], [32]. The minimum pearson's correlation values between replicates in each group were between 0.93 and 0.95 signifying good reproducibility. The statistical analysis for detecting the global differences in the gene expression between the groups was carried out using Bioconductor's Limma package [33]. For filtering out the differentially expressed genes, the minimum fold-change limit was set at 1.5 and the significance level at 0.01. Ingenuity Pathway analysis (http://www.ingenuity.com/) was used to study HIF1α pathway.

### RT-PCR

RT-PCR analysis was carried out as previously described [34]. The results were normalized with the expression values of housekeeping gene EF1α. The primer and probe sequences (File S1).

### Flow Cytometry

The morphology of the colonies was observed with Stereo LuMAR V.12 stereomicroscope (Zeiss).

Cells were harvested from the feeder free plates with 0.01% Trypsin-EDTA and washed twice with cold buffer (D-PBS, 2% FCS, 0.01% sodium azide). Primary antibodies (File S1) were incubated for 30 min at +4°C, after which cells were washed. Secondary antibodies were incubated for 30 min at +4°C. The cells were washed and run with FACS Calibur (BD Biosciences) and analyzed with Cyflogic Version 1.0.2 (Perttu Terho CyFlo Ltd).

### RNA Interference and hESC Transfection

RNA interference was performed with synthesized siRNA oligonucleotides (Sigma) or (MWG-Biotech) using previously validated siRNAs targeting HIF1α and HIF2α (File S1). Transfections were performed according to the manufacturer’s protocols using Lipofectamine 2000 reagent (Invitrogen) with 200 nM concentration of siRNAs. HIF siRNAs were introduced to hESCs by two consecutive transfections performed at 24 h and 48 h after plating in normoxic conditions. After 48 h from the first transfection cells were introduced to hypoxia where overnight balanced hypoxic media was introduced immediately.

## Results

### Hypoxic Response in hESCs

**Figure 1:**
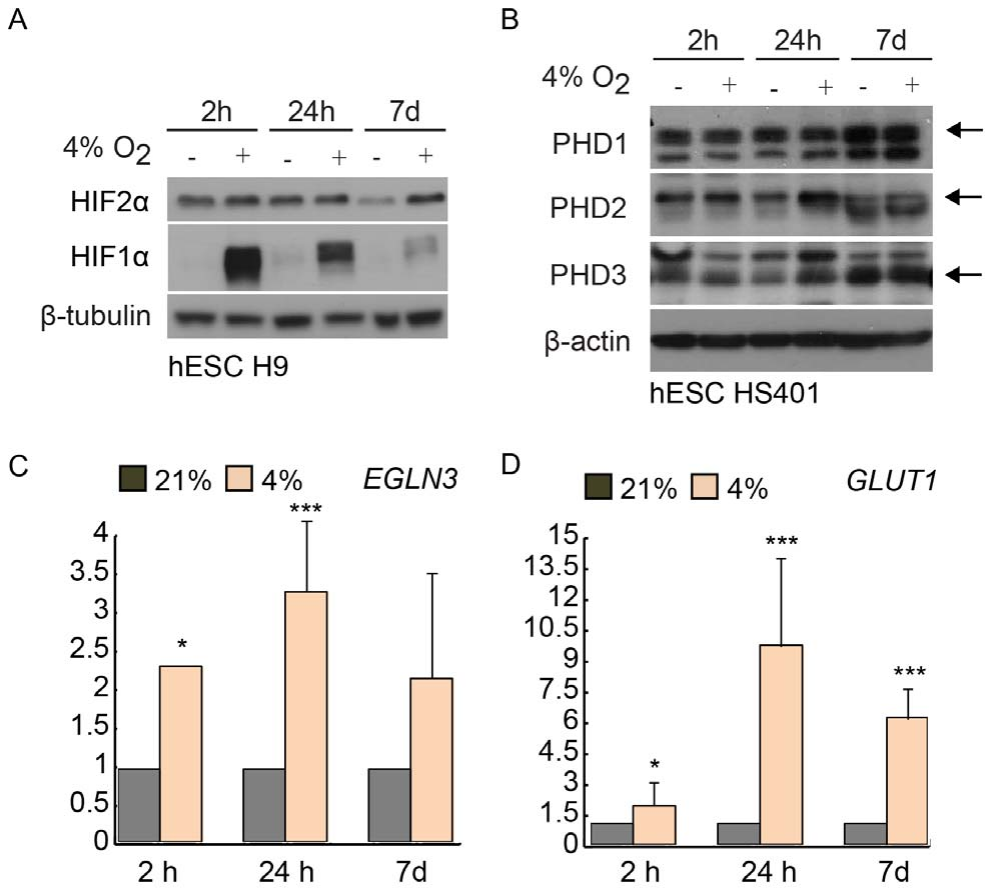
Hypoxia response in human embryonic stem cells. A) Western blot analysis of HIF1α and HIF2α. B) Western blot analysis of PHD1, PHD2 and PHD3. C) RT-PCR validation of *EGLN3* (PHD3). D) RT-PCR validation of *GLUT1*. * = p<0.05, *** = p<0.001. RT-PCR data from two replicate cultures of hESC line H9.

H9, HS401 and HS360 hESC lines were exposed to normoxia (21% O_2_) and hypoxia (4% O_2_) for 2 hours, 24 hours or 7 days. All incubations and daily changes of culture medium were performed under strictly controlled atmospheric conditions. Importantly, cells cultured under hypoxic conditions were not exposed to ambient oxygen at any stage of the experiment. Protein analyses showed that HIF1α protein was stabilized after short (2 h) hypoxic exposure. However, the level of HIF1α protein was reduced already after intermediate (24 h) culture in hypoxia and was almost undetectable after prolonged (7 days) hypoxic culture, whereas HIF2α was expressed in all conditions and time points analyzed (Figure 1A). Interestingly, all HIF prolyl hydroxylase isotypes (PHD1–3) were expressed already in normoxic conditions. Hypoxia further increased the amount of PHD2 and PHD3 at 24 h timepoint in all hESC lines studied (Figure 1B). To further validate the experimental set up we analyzed the mRNA levels of known hypoxia-regulated genes, *EGLN3* (PHD3) and *GLUT1* with quantitative RT-PCR (Figure 1C, D). As expected, both genes showed transcriptional activation in response to low oxygen in all cell lines.

### Hypoxia Enriches SSEA-3 Positive Cells and Reduces Levels of TRA-2-54

**Figure 2:**
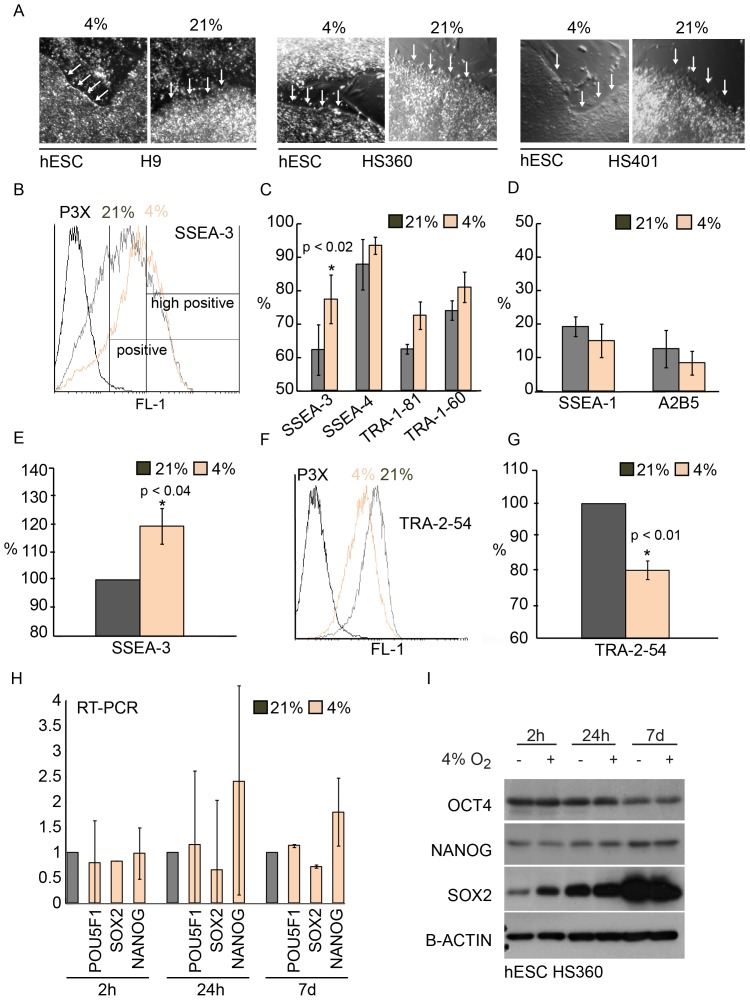
Morphology and expression of pluripotency markers. A) Morphology of hypoxia (4%) and normoxia (21%) cultured colonies after 7 days of culture for each hESC line (H9, HS360 and HS401) respectively. B–G) Flow cytometry analysis performed after 7 day culture. B) A histogram showing SSEA-3 positive cells in hypoxia and normoxia versus negative control P3X and gating example for positive and high positive populations. C) Proportion of positive cells expressing pluripotency surface antigens SSEA-3, SSEA-4, TRA-1-81, and TRA-1-60. D) Proportion of positive cells expressing differentiation surface antigens SSEA-1 and A2B5. E) Proportion of high positive cells expressing SSEA-3. F) Histogram of TRA-2-54 expressing cells. G) Proportion of high positive cells expressing TRA-2-54 (alkaline phosphatase). H) Real-time PCR analysis of *POU5F1* (*OCT4*), *SOX2* and *NANOG* in response to hypoxia (4%) over time (2 h, 24 h, and 7 d) compared to normoxia (21%). Data from two replicate cultures of hESC line H9. I) Western blot analysis of protein levels of OCT4, NANOG and SOX2 in hypoxia over time. B-ACTIN was used as a loading control.

To assess the effects of hypoxia on the phenotypic features of hESC lines, we recorded the morphological changes of colonies cultured 7 days in 4% or 21% oxygen. Typical for less differentiated state the edges of the cell colonies grown in hypoxia were well defined and sharper compared to normoxic cultures (Figure 2A). To further characterize the differentiation status, we analyzed the expression of cell surface markers with flow cytometry. The pluripotency markers SSEA-4, TRA-1-81, TRA-1-60 and especially SSEA-3 were all enriched in hypoxic cells whereas differentiation markers, A2B5 and SSEA-1 were decreased (Figure 2B–E). In contrast to other pluripotency markers, expression of TRA-2-54 (alkaline phosphatase) was clearly reduced in hypoxia. The histogram analysis revealed that hypoxia cultured cells were positive for TRA-2-54 (Figure 2F), but the proportion of highly positive cells for TRA-2-54 was significantly lower than in cells cultured in normoxia (Figure 2G).

### Stable Expression of Core Pluripotency Factors *OCT4*, *NANOG* and *SOX2*

*OCT4*, *NANOG* and *SOX2* are the core transcription factors regulating hESC pluripotency and differentiation [35], [36]. To study whether their expression is directly regulated by oxygen at transcriptional level we analyzed the mRNA levels with quantitative RT-PCR (2 h, 24 h and 7d) from normoxic and hypoxic cultures. Statistically significant differences were not detected, implying that these factors are not oxygen-regulated (Figure 2H). Interestingly, while Western blot analysis revealed stable expression of OCT4 and NANOG at protein level, SOX2 protein levels increased steadily along culturing and showed hypoxia responsiveness after immediate exposure to low oxygen (2h) (Figure 2I).

### Transcriptome of Hypoxia Cultured Cells

To identify the hypoxia-induced changes in the global gene expression profiles, transcriptomes of all three hESC lines cultured in ambient (21%) and low oxygen (4%) atmospheres for 2 hours, 24 hours and 7 days were analyzed with Affymetrix GeneChip Human Exon 1.0 ST Arrays. The data from all three cell lines was combined to avoid cell line-specific biases and to have as representative biological replicates as possible.

**Figure 3:**
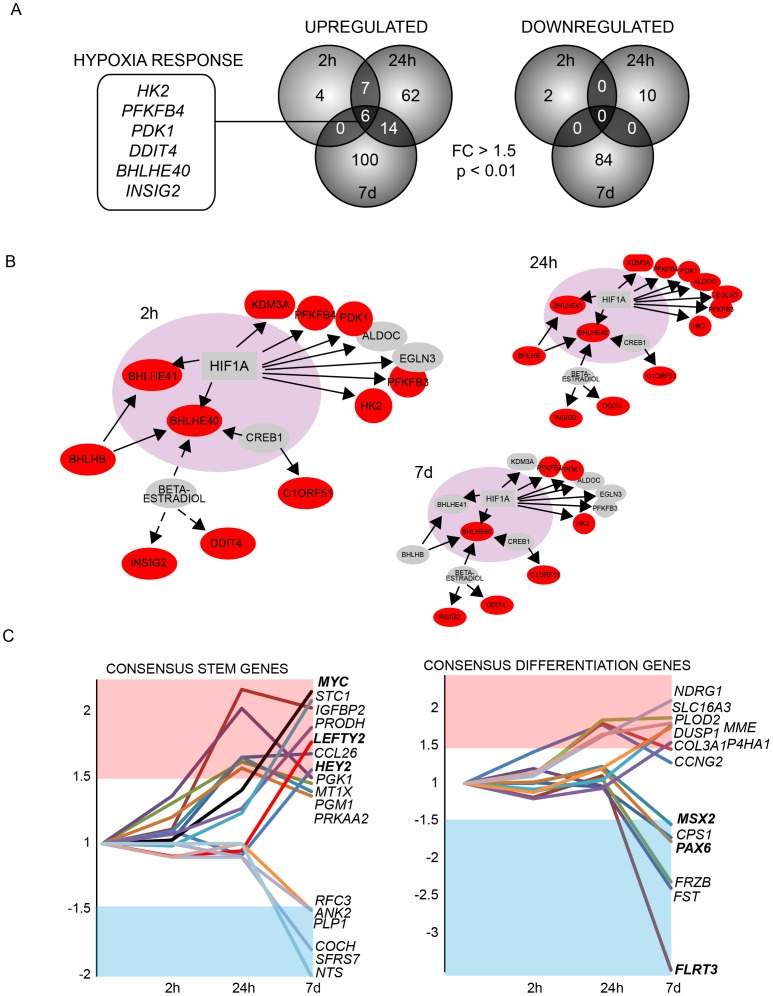
Hypoxia activated transcriptome. A) Number of statistically significantly (p<0.01, Fold change >1.5) upregulated and downregulated genes detected at various time points (2h, 24h, 7d) in response to hypoxia. Data from three hESC lines analysed with GeneChip Human Exon 1.0 ST Array (Affymetrix). Six hypoxia-responsive genes were upregulated in response to hypoxia in each cell line and at all time points analysed. B) Responsiveness of HIF1α activated genes at each timepoint in the GeneChip Human Exon 1.0 ST Array data. Upregulated targets are shown in red, line (direct targets), dashed line (indirect targets). Figure based on Ingenuity Pathway analysis (http://www.ingenuity.com/). C) Expression of a panel of genes differentially expressed between hypoxia and normoxia. The genes are classified to those characteristic for consensus hESC or differentiation stage signatures [42].

When only significant changes (p<0.01 and absolute fold change >1.5) were taken into account a list of 193 up regulated and 97 down regulated transcripts were detected in hypoxia (Figure 3A, Table 1, Table S1). Six of these genes were up regulated in all cell lines and at each time point studied. Importantly, all of them were well characterized hypoxia-responsive genes; *HK2*, *PFKFB4* [37], *PDK1* [38], *DDIT4* (*REDD1*) [39], *BHLHE40* (*DEC1*) [40] and *INSIG2* [41]. To further study the hypoxic regulation in hESCs, we performed the Ingenuity Pathway analysis (http://www.ingenuity.com/) (Figure 3B). An induction of HIF1α responsive genes was detected already after 2 hours, being most prominent at 24 hours. In correlation with declining HIF1α protein levels (Figure 1A), only few HIF-regulated transcripts were detected after prolonged hypoxic culture (7 days) (Figure 3B).

**Table 1:** Top ten transcripts differentially expressed in hypoxia compared to normoxia at different timepoints.

timepoint	upregulated	Fold Change	P value	downregulated	Fold Change	P value
*2h*	*DDIT4*	3.46	2.26E-05	*ANKRD1*	−2.23	0.006601
*2h*	*BHLHE40*	2.18	0.00025	*LOC100127909*	−1.828	0.006433
*2h*	*INSIG2*	2.15	0.002651	–	–	–
*2h*	*ARRDC3*	2.15	0.001489	–	–	–
*2h*	*PFKFB3*	2.10	0.0002	–	–	–
*2h*	*JMJD1A*	2.03	0.001043	–	–	–
*2h*	*PDK1*	1.92	0.00302	–	–	–
*2h*	*BHLHE41*	1.83	0.004617	–	–	–
*2h*	*PFKFB4*	1.79	0.003491	–	–	–
*2h*	*HK2*	1.77	0.000272	–	–	–
*24h*	*NRN1*	4.24	9.01E-05	*LOC100128170*	−2.35	0.006674
*24h*	*PDK1*	2.83	0.000054	*LOC100132938*	−2.00	0.009072
*24h*	*BNIP3*	2.78	0.002063	*LOC100129540*	−1.98	0.003155
*24h*	*INSIG2*	2.63	0.000416	*LOC401913*	−1.92	0.001397
*24h*	*DDIT4*	2.58	0.000319	*LOC100131505*	−1.91	0.007727
*24h*	*LOC729046*	2.51	0.000789	*LOC100133208*	−1.66	0.006312
*24h*	*JMJD1A*	2.48	0.000112	*FAM45A*	−1.64	0.000753
*24h*	*C4orf47*	2.47	0.000405	*RPL14P1*	−1.60	0.002758
*24h*	*RP13-36C9.6*	2.43	0.000368	*LOC100132310*	−1.54	0.007138
*24h*	*PFKFB4*	2.40	0.000113	*OR8I2*	−1.52	0.008112
*7d*	*LOC100132782*	2.93	0.0001473	*FLRT3*	−1.82	1.89E-06
*7d*	*DDIT4*	2.69	0.0002157	*LOC728715*	−1.49	0.000535
*7d*	*LOC100128019*	2.57	0.0047926	*FST*	−1.28	5.99E-05
*7d*	*RPS24P9*	2.35	0.0033802	*FRZB*	−1.22	0.004472
*7d*	*BHLHE40*	2.30	0.0001346	*IFRG15*	−1.22	0.001592
*7d*	*INSIG2*	2.30	0.00144	*SNORD116-28*	−1.12	0.001074
*7d*	*SNORD115-7*	2.29	0.0003424	*SOAT1*	−1.12	0.000156
*7d*	*IL8*	2.28	0.0001809	*KTELC1*	−1.09	0.001621
*7d*	*ADCY8*	2.23	1.051E-05	*MIRHG1*	−1.06	0.006898
*7d*	*LOC100129775*	2.23	0.0026457	*RSPO3*	−1.04	0.004747

Data from three hESC lines analysed with GeneChip Human Exon 1.0 ST Array (Affymetrix).

To clarify the mechanisms how hypoxic growth conditions contribute to pluripotency and differentiation of hESCs, we surveyed the transcriptome data for all transcripts associated with regulation of hESC physiology. Importantly, 12% of all oxygen-regulated transcripts detected were linked to mechanisms controlling pluripotency and differentiation [42] (Figure 3C). The majority of transcriptional responses were detected in prolonged hypoxic culture. From these genes of particular interest are HEY2 with a role in notch signaling [43], LEFTY2 involved in TGFβ-signaling [44] and IGFBP2 which is overexpressed in the stem cell compartment of glioblastomas promoting proliferation and survival of brain tumors [45]. Moreover, the levels of MYC that contributes to induction and maintenance of pluripotency [21]–[24] were elevated.

Hypoxic culture conditions also modified the expression of genes driving cell differentiation (Figure 2C), further supporting an idea that hypoxia directly influences the differentiation potential of cells. Among these genes were neuroectoderm marker PAX6 [46], trophoblast and placenta associated transcription factor MSX2 [47], and a primitive streak marker FLRT3 [48], which all showed less expression in response to prolonged hypoxic culturing compared to normoxic cultures.

### Hypoxic Culturing Enhances Expression of MYC

**Figure 4:**
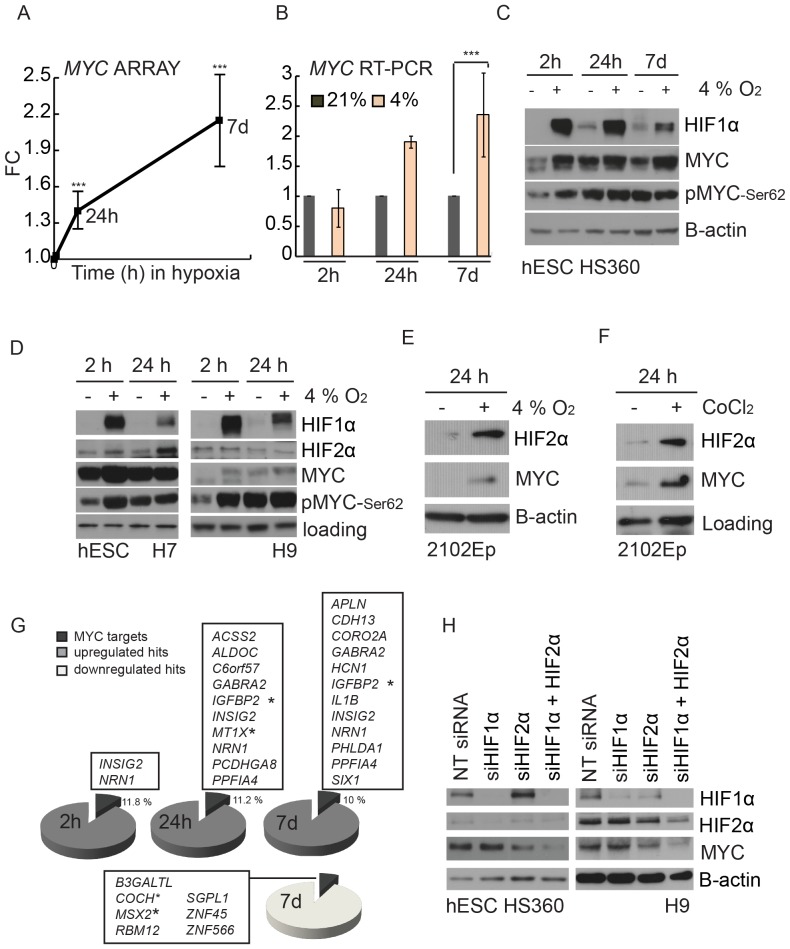
Induction of MYC in hypoxic culture. A) The expression of *MYC* in the exon-array data. Fold change (FC) compared to normoxic culture, *** = p<0.005. B) RT-PCR validation of the *MYC* expression in hESCs, *** = p<0.001. Data from two replicate cultures of hESC line H9. C) Western blot analysis of MYC, pMYC-Ser62 and HIF1α in hESC line HS360. D) Western blot analysis of HIF1α, HIF2α, MYC and pMYC-Ser62 in hESC lines H7 and H9 (individual experiments). E) Western blot analysis of HIF2α and MYC in hypoxia (4%) and F) in chemically induced (CoCl_2_) hypoxic conditions in embryocarcinoma cell line 2102Ep. G) Transcripts identified to change in response in to hypoxia in hESCs (exon array data) and reported to have MYC binding site in hESCs [23]. * = transcripts shared with the data presented on Figure 3C. H) Western blot analysis of HIF1α, HIF2α and MYC after small interfering (si) silencing (NT =  negative control siRNA). Cells treated 48 h with siRNA before exposure to hypoxia for 2 h.

MYC regulates a wide range of cellular processes and aberrant expression of MYC leads to dramatic changes in cellular homeostasis. In addition to sustaining cellular growth and proliferation, MYC has direct effects on pluripotency and self-renewal [21]–[24]. *MYC* mRNA was significantly elevated already after 24 hours and was further increased after 7 days in hypoxia (Figure 4A). Elevated expression of *MYC* was validated with quantitative RT-PCR (Figure 4B).

Western Blot analysis detected induction of MYC protein after short (2h) hypoxic culture (Figure 4C). The increase in the total MYC protein expression correlated directly with the amount of phosphorylated MYC (MYC-Ser62) (Figure 4C,D), the modification known to protect MYC protein from proteosomal degradation [49]. At later time points (24 h and 48h) there was no detectable difference in the MYC-Ser62 levels between hypoxia and normoxia. However, the total MYC protein levels remained elevated in all hypoxic timepoints (Figure 4C) in accordance with the transcriptional induction of *MYC* gene (Figure 4A,B). This suggests that the immediate responses to decreased oxygen levels are mediated via protein stabilization and prolonged responses by the increase in gene transcription.

Since MYC is a well characterized oncogene, we wanted to investigate if hypoxia upregulates expression of MYC also in pluripotent cancer cells. For these studies we used embryonal carcinoma cells 2102Ep (ECs) that resemble hESCs. The hypoxia-induced stabilization of HIF1α and effect on pluripotency markers were comparable to those in hESCs (Figure S1A,B). As a result, expression of MYC protein was induced in 2102Ep EC cells in response to hypoxic culture as well as to chemical hypoxia mimetic CoCl_2_ (Figure 4E, F).

To assess the magnitude of direct effects of hypoxia-regulated MYC expression on the hypoxia-mediated alterations in global gene expression profiles, we overlaid our data with published MYC Chip-chip [23]. Our analysis revealed that over 10% of the hypoxia-induced genes in our data set have a MYC binding site in their regulatory regions (Figure 4G). This suggests that induction of MYC in hypoxia treated hESCs has influenced the expression of its specific target genes.

To study if hypoxic MYC expression is dependent on HIF factors, we performed individual and simultaneous silencing of HIF1α and HIF2α. As a result, MYC expression was clearly affected upon HIF2α silencing whereas silencing of HIF1α had no effect on MYC levels (Figure 4H). Based on these results we conclude that high MYC expression in hypoxia is dependent on HIF2α expression.

## Discussion

Low oxygen conditions contribute to maintenance of the pluripotent state of hESCs as well as cellular differentiation. To investigate the potential mechanisms and regulatory networks underlying these processes we carried out carefully controlled experiments with three different hESC lines and one embryonal carcinoma cell line. Crucial for our experimental approach was to have a complete control over the atmospheric conditions in which the cells were cultured. Special care was taken to make sure that the hypoxia treated cells were not exposed to ambient oxygen at any stage of our experimentation. Furthermore, in our analysis we merged data from three distinct hESC lines. This approach enabled us to filter out all cell line-specific effects and extract the core hESC response to hypoxia.

Expression of SSEA-3 was increased when hESCs were cultured in continuous hypoxia (Figure 2C, p<0.02). SSEA-3 is a surface marker which decreases rapidly in response to differentiation [50]. Importantly, the difference detected was nearly 20% between hypoxic and normoxic cultures, which is unexpectedly high, considering that silencing of key pluripotency transcription factors OCT4, SOX2 and NANOG that leads to rapid loss of pluripotency and differentiation have shown to induce comparable decrease in the expression of SSEA-3 [51], [52]. Although recent papers have shown that hESCs morphologically resemble less differentiated cell stage in hypoxia, no differences have been reported in the expression of SSEA-4, SSEA-1, TRA-1-60 and TRA-1-81 [4], [6], [53]. Similarly, we were not able to observe any statistical difference in the expression of SSEA-4, TRA-1-81 or TRA-1-60, although there was a consistent trend for increased levels of these indicators of undifferentiated cell stage. Consistently, expression of differentiation markers A2B5 and SSEA-1 was decreased. On the contrary to other surface markers, the expression of TRA-2-54, a marker for alkaline phosphatase, was decreased in hypoxia [54]. Similarly, Chen et al. detected decreased alkaline phosphatase staining in hESCs cultured in low oxygen conditions [53]. Actually, hypoxia has been reported to inhibit alkaline phosphatase activity in rat calvarial osteoblast [55]. Therefore, the diminished expression of alkaline phosphatase could be a direct consequence of hypoxia response rather than an implication of a decreased pluripotent state.

To our knowledge this is the first report to show the regulation of Hif prolyl hydroxylases (PHD1–3) in hESCs. In various cell models tested both PHD2 and PHD3 have been shown to be strictly oxygen regulated being induced in response to hypoxia whereas PHD1 has more stable expression despite variations in oxygen conditions. Interestingly, our transcriptomics as well as Western blot data from three hESC lines showed that hESCs express all PHD isoforms already in ambient oxygen conditions. The expression of PHD2 and PHD3 was further increased in hypoxia. This may partially explain the transition from HIF1α to HIF2α in hESCs after prolonged hypoxia, since prolyl hydroxylases have different activity towards HIFα subunits. PHD2 has more effect on HIF1α than HIF2α, whereas PHD3 is clearly more efficient regulator of HIF-2α [56]. Particularly PHD3 controls the HIF-activity also in hypoxia and fine-tune the HIF activity to maintain gene activation in appropriate levels [57]. Recently it has become evident that PHDs directly control cell differentiation. PHD1 and PHD3 are required for functional erythropoiesis in adult mice [58], PHD3 also regulates differentiation of skeletal muscle cells through controlling the activity of NF-KappaB dependent signalling [59], [60]. It remains to be investigated what are the targets and molecular mechanisms that take place in addition to HIF regulation to control the differentiation of hESCs. It is likely that besides HIFs additional hydroxylation and interaction partners participate in this regulation. Interestingly, PHD3 was recently shown to enhance G_1_ to S transition and hence survival of carcinoma cells in hypoxia [61]. As hESCs are characterized by short G_1_-phase [62]–[64], PHD3 may support G_1_ to S transition and survival of hESCs in hypoxic conditions.

Recent studies have clearly established the differential regulation and physiological roles for HIF1α and HIF2α [65]. It has been shown that HIF1α is degraded and HIF2α stabilized after long-term hypoxic culture of hESCs [6], [66]. Similarly, we detected HIF1α-mediated hypoxia response in the early time points, which was effectively quieted in long-term hypoxia. These results showing temporary exclusive expression patterns for HIF1α and HIF2α suggest different regulatory mechanisms and roles for HIF1α and HIF2α in hESCs. The stabilization of HIF2α was not consistent between cell lines at early timepoints studied (Figure 1A, 4D). Interestingly, Forristal et al. have shown that in hESCs HIF2α is mainly cytoplasmic after 48h of exposure and nuclear only after a long-term exposure to 5% oxygen [6]. Therefore, analysis of the total HIF2α levels can fail to detect the nuclear induction of HIF2α which may explain the inconsistence seen between the cell lines. Interestingly, hypoxia induced HIF1α has been shown to inhibit MYC activity on its target genes whereas HIF2α can stimulate MYC activity in certain cell types [67], [68]. In addition, overexpression of HIF2α in endothelial cells results in increased MYC protein expression [69] and HIF2α has been shown to enhance expression of MYC and promote tumor growth in renal clear cell carcinoma lines [67]. Our results support these findings as HIF2α expression and loss of HIF1α correlates with elevated transcription of *MYC*. Further, we have shown here that MYC expression in hypoxia is dependent on HIF2α, but not HIF1α.

Consistent with the earlier reports [4], [53], [66], [70], we were not able to detect any significant changes in expression of *OCT4*, *SOX2* or *NANOG*. This implies that hypoxia influences the physiology of hESCs through other parallel signaling cascades. Here we report that hypoxia cultured hESCs express elevated levels of MYC. This may explain, at least partly, the mechanism behind the observed changes in hypoxic cultured hESCs. Recently it was shown that MYC protein levels decrease during the differentiation and that MYC inhibitor 10058-F4, by disturbing the formation of functional MYC/MAX heterodimer, leads to differentiation and induction of germ layer markers in hESCs [22]. In support, MYC knockdown by lentiviral sh-RNAs leads to growth arrest and up-regulation of differentiation-associated transcripts in hESCs [23]. These studies have clearly established the vital role for MYC in maintaining the pluripotency and growth. We did not detect difference in the cell numbers or RNA amounts measured after prolonged hypoxia and normoxia samples (Figure S1C,D), implying that elevated MYC levels mainly affect the differentiation status of hESCs rather than growth.

Unpredictably, overexpression of chimeric c-MycER protein in hESCs leads to apoptosis, decrease in *OCT4* and *NANOG* expression and induction of endodermal (*GATA4* and *GATA6*) and trophectodermal (*CDX2* and *CGA*) genes [71]. On the contrary, we report that hypoxia induced endogenous overexpression of MYC does not affect expression of core pluripotency factors or increase *GATA4, GATA6, CDX2* or *CGA* expression. Based on our results, endogenous induction of MYC increases the portion of SSEA-3 expressing cells and subsequently decreases the expression of a set of genes tightly linked to differentiation such as *PAX6*, *FLRT3* and *MSX2*. This supports the hypothesis of MYC having an important role in the regulation of hESC pluripotency.

Interestingly, hESC lines derived in hypoxia maintain two active X chromosomes, which is considered as a ground state of pluripotency [7]. The gene expression profiles of these cell lines revealed that expression of *MYC* decreased when cells were exposed to normoxia. Combining this and the data we present here, it seems that hESCs which have been cultured under physiological levels of oxygen have increased levels of *MYC* which is likely to have an important role in regulating ground state of cellular pluripotency.

What are the mechanisms leading to stabilization of MYC in hypoxia-treated hESCs? Phosphorylation on Ser62 by extracellular-regulated kinase 1,2 (ERK) stabilizes MYC through preventing its proteosomal degradation [49]. High ERK activity has been reported to promote self-renewal of hESCs [72]. Interestingly, MYC-Ser62 phosphorylation is reversed by protein phosphatase 2A (PP2A), which is critical for maintaining the self-renewal of hESCs [22]. ERK mediated MYC phosphorylation is also activated by oxidative stress in tumor cells [73]. Thereby, the immediate MYC stabilization at protein level after exposure to hypoxia reported in this study is likely to be regulated by activation of ERK signaling.

Taken together, we report here an increase in MYC expression in hESCs cultured in hypoxia which is dependent on HIF2α. In agreement with this, HIF2α is stabilized after the cells have been cultured in hypoxia for a longer period (7d) which may in turn promote MYC function. Moreover, the observed endogenous induction of MYC in hypoxia may explain why reprogramming is enhanced in hypoxic culture conditions, since MYC overexpression is known to increase reprogramming efficiency. Hypoxic induction of HIF2α, PHD3 and MYC, together with stable expression of OCT4, NANOG and SOX2 and increased expression of SSEA-3 suggests that induction of MYC in hypoxia supports the maintenance and pluripotency of hESCs.

## Supporting Information

Figure S1 A) Western blot analysis of HIF1α, PHD1, PHD2, and PHD3 in 2102Ep cell line. B) Western blot analysis of OCT4, NANOG, and SOX2 in 2102Ep cell line. C) Number of cells and D) Total RNA extracted after 7 day cultures in normoxia and hypoxia (data from six replicate cultures of hESCs).
(TIF)

Table S1 **Significant gene expression changes of hESC lines cultured in (21%) or (4%) oxygen for 2 h, 24 h or 7 days.** Affymetrix GeneChip Human Exon 1.0 ST Arrays.
(XLSX)

File S1 **List of primers, antibodies and siRNAs used in the study.**
(DOCX)
