# Supplementary figures and images for: Continuous Hypoxic Culturing of Human Embryonic Stem Cells Enhances SSEA-3 and MYC Levels
Source: PLoS One. 2013 Nov 13;8(11):e78847. doi: 10.1371/journal.pone.0078847 (PMC3827269; doi:10.1371/journal.pone.0078847)

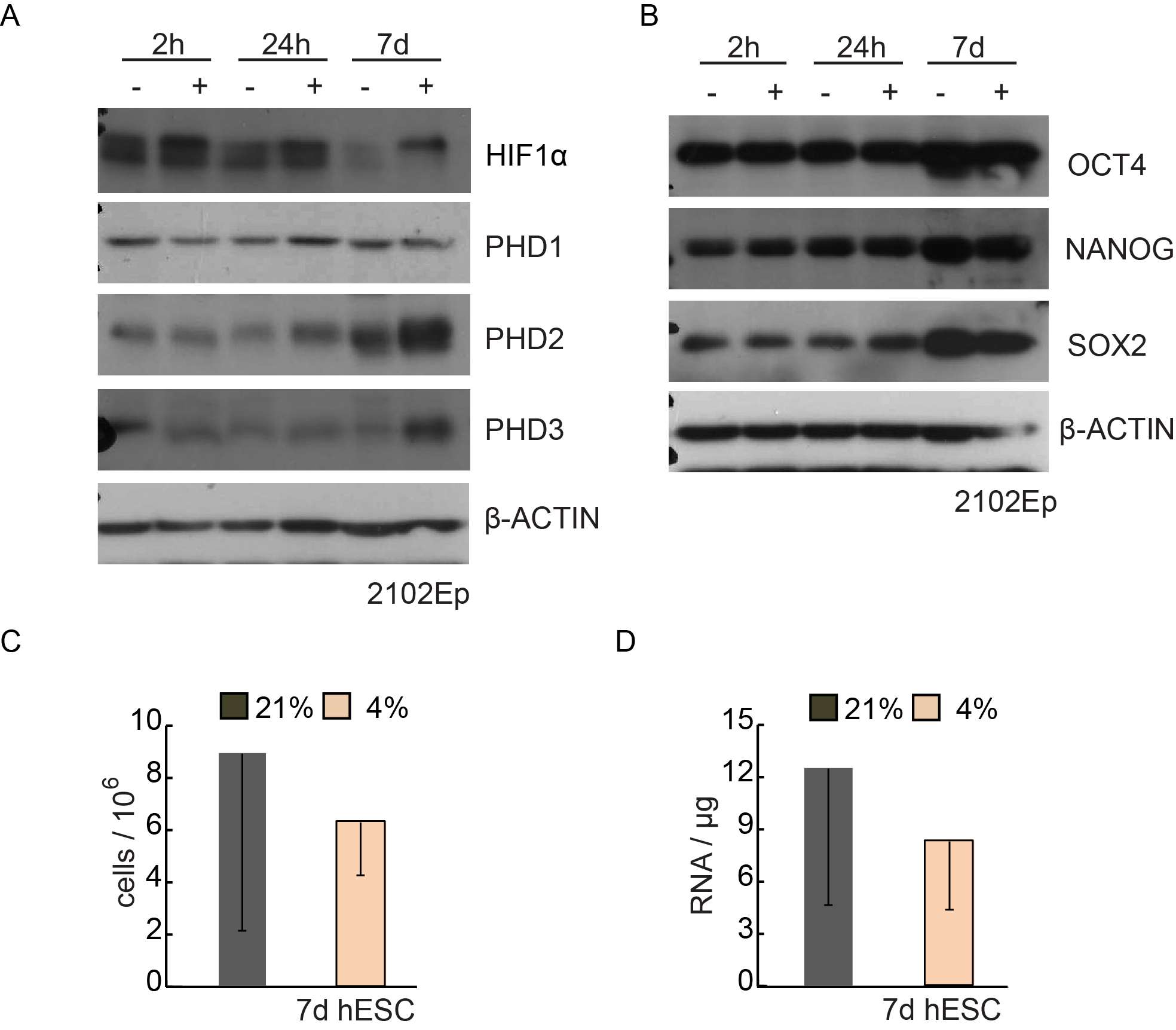

Supplement: Figure S1 — A) Western blot analysis of HIF1α, PHD1, PHD2, and PHD3 in 2102Ep cell line. B) Western blot analysis of OCT4, NANOG, and SOX2 in 2102Ep cell line. C) Number of cells and D) Total RNA extracted after 7 day cultures in normoxia and hypoxia (data from six replicate cultures of hESCs). (TIF) [file pone.0078847.s001.tif]
