# Supplementary material for: Continuous Hypoxic Culturing of Human Embryonic Stem Cells Enhances SSEA-3 and MYC Levels
Source: PLoS One. 2013 Nov 13;8(11):e78847. doi: 10.1371/journal.pone.0078847 (PMC3827269; doi:10.1371/journal.pone.0078847)
Supplement: File S1 — List of primers, antibodies and siRNAs used in the study. (DOCX) [file pone.0078847.s003.docx]

File S1: (Närvä & Pursiheimo)

| **Target** | **Primer forward 5’-3’** | **Primer reverse 5’-3’** | **Probe** |
| --- | --- | --- | --- |
| *MYC* | CACCAGCAGCGACTCTGA | GATCCAGACTCTGACCTTTTGC | 34 (Roche) |
| *EGLN3* | CGAAGTGCAGCCCTCTTACG | TTTTGGCTTCTGCCCTTTCTT | 61 (Roche) |
| *GLUT1* | GTGGGCATGTGCTTCCAGTA | AAGAACAGAACCAGGAGCACAGT | 67 (Roche) |
| *OCT4* | AGCAAAACCCGGAGGAGT | CCACATCGGCCTGTGTATATC | 35 (Roche) |
| *NANOG* | CCTGAACCTCAGCTACAAACAG | GCTATTCTTCGGCCAGTTGT | 87 (Roche) |
| *SOX2* | ATGGGTTCGGTGGTCAAGT | GGAGGAAGAGGTAACCACAGG | 19 (Roche) |

| **Target protein** | **Antibody** | **Company** | **Dilution** |
| --- | --- | --- | --- |
| HIF1α | 610959 | BD Transduction Laboratories | 1:3000 |
| β-actin | A5441 | Sigma-Aldrich | 1:10 000 |
| PHD1 | NB 100-310 | Novus Biologicals | 1:1000 |
| PHD2 | NB 100-137 | Novus Biologicals | 1:3000 |
| PHD3 | NB 100-139 | Novus Biologicals | 1:2000 |
| HIF2α | NB-100-122 | Novus Biologicals | 1:200 |
| MYC | NB600-302 (9E10) | Novus Biologicals | 1:200 |
| pMYC-Ser62 | 71-161 | Bio Academia | 1:1000 |
| OCT4 | sc-9081 | Santa-Cruz Biotechnology | 1:500 |
| NANOG | sc-33759 | Santa-Cruz Biotechnology | 1:500 |
| NANOG | AF1997 | R&D Systems | 1:1000 |
| SOX2 | MAB2018 | R&D Systems | 1:1000 |
| GAPDH | 5G4 | HyTest Ltd | 1:20 000 |
| Anti-rabbit-HRP | 554021 | BD Pharminged | 1:10 000 |
| Anti-mouse-HRP | sc-2005 | Santa-Cruz Biotechnology | 1:10 000 |

| **Target** | **Antibody** | **Company** | **Dilution** |
| --- | --- | --- | --- |
| P3X | Sheffield | Gift from P.W.A | 1:50 |
| SSEA-3 | Sheffield | Gift from P.W.A | 1:50 |
| SSEA-4 | Sheffield | Gift from P.W.A | 1:50 |
| TRA-1-81 | Sheffield | Gift from P.W.A | 1:50 |
| TRA-1-60 | MAB4360 | Millipore | 1:100 |
| SSEA-1 | Sheffield | Gift from P.W.A | 1:50 |
| A2B5 | Sheffield | Gift from P.W.A | 1:50 |
| TRA-2-54 | Sheffield | Gift from P.W.A | 1:50 |
| Alexa-488 IgG | A11001 | Invitrogen | 1:200 |
| FITC anti-mouse IgG + IgM | M30801 | Caltag Laboratories | 1:150 |

| **siRNA target** | **sequence** | **Reference** |
| --- | --- | --- |
| non-target control | 5’-CCUACAUCCCGAUCGAUGAUG-3’ | Berra et al. 2003 |
| HIF1α | 5’-AACUAACUGGACACAGUGUGU(dTdT)-3’ | Marxsen et al. 2004 |
| HIF2α | 5’-GCGACAGCUGGAGUAUGAAUU(dTdT)-3’ | Warnecke et al. 2004, Larsen et al. 2012 |

**References:**

Berra E, Benizri E, Ginouves A et al. HIF prolyl-hydroxylase 2 is the key oxygen sensor setting low steadystate levels of HIF-1alpha in normoxia. EMBO J 2003;22:4082-4090.

Marxsen JH, Stengel P, Doege K, Heikkinen P, Jokilehto T, Wagner T, Jelkmann W, Jaakkola P, Metzen E. [*Hypoxia-inducible factor-1 (HIF-1) promotes its degradation by induction of HIF-alpha-prolyl-4-hydroxylases.*](http://www.ncbi.nlm.nih.gov/pubmed/15104534) Biochem J. 2004 Aug 1;381(Pt 3):761-7.

[Warnecke C](http://www.ncbi.nlm.nih.gov/pubmed?term=Warnecke%20C%5BAuthor%5D&cauthor=true&cauthor_uid=15240563), [Zaborowska Z](http://www.ncbi.nlm.nih.gov/pubmed?term=Zaborowska%20Z%5BAuthor%5D&cauthor=true&cauthor_uid=15240563), [Kurreck J](http://www.ncbi.nlm.nih.gov/pubmed?term=Kurreck%20J%5BAuthor%5D&cauthor=true&cauthor_uid=15240563), [Erdmann VA](http://www.ncbi.nlm.nih.gov/pubmed?term=Erdmann%20VA%5BAuthor%5D&cauthor=true&cauthor_uid=15240563), [Frei U](http://www.ncbi.nlm.nih.gov/pubmed?term=Frei%20U%5BAuthor%5D&cauthor=true&cauthor_uid=15240563), [Wiesener M](http://www.ncbi.nlm.nih.gov/pubmed?term=Wiesener%20M%5BAuthor%5D&cauthor=true&cauthor_uid=15240563), [Eckardt KU](http://www.ncbi.nlm.nih.gov/pubmed?term=Eckardt%20KU%5BAuthor%5D&cauthor=true&cauthor_uid=15240563). *Differentiating the functional role of hypoxia-inducible factor (HIF)-1alpha and HIF-2alpha (EPAS-1) by the use of RNA interference: erythropoietin is a HIF-2alpha target gene in Hep3B and Kelly cells.* [FASEB J.](http://www.ncbi.nlm.nih.gov/pubmed/15240563) 2004 Sep;18(12):1462-4. Epub 2004 Jul 1.

Larsen H, Muz B, Khong T L, Feldmann M & Paleolog E M. *Differential effects of Th1 versus Th2 cytokines in combination with hypoxia on HIFs and angiogenesis in RA.* Arthritis Research & Therapy 2012, 14:R180
